# Books

**Published:** 1882-01

**Authors:** 


### Books.

Transactions of the Mississippi State Medical Association, at the Fourteenth Annual Session, held at Winona, April 6, 7 and 8, 1881. B. F. Ward, M.D., Winona, President; Wirt Johnson, M.D., Jackson, Secretary.

The regular publication of the proceedings of State societies in a volume of transactions is a wise and useful practice. The annual volume serves to stimulate the members to more active endeavor, to unify the organization, to record its history and to mark its progress.

The volume before us, besides the daily proceedings of the body, contains the address of the retiring president, Dr. W. F. Hyer, Hudsonville, on "The Science of Medicine an Important Department of the Science of Humanity;" an address by Hon. J. S. Morris, of Vicksburg, on "The Rights, Duties and Responsibilities of Physicians Before the Courts;" "Abortive Treatment of Pneumonia," by Dr. W. Y. Gadbury, Yazoo City; "New Remedies," by Dr. W. A. Vaughn, Columbus; "Diphtheria," by Dr. J. B. Gresham, West Point; "Recent Advances in General Pathology," and "Diseases of the Gastro-Enteric Membrane in Infancy and Childhood," by Dr. B. F. Ward, Winona; "Report on Surgery," by Dr. M. C. Craft, Jackson; and "A New Appliance for Fracture of the Lower Extremity," by Dr. W. Y. Gadbury, Yazoo City.

The list of members, while not as long as it ought to be, would seem to indicate that the Association is fairly representative of the profession of the State, and the proceedings and papers evidence a worthy interest in the organization on the part of the members.

---

TRANSACTIONS OF THE MICHIGAN STATE MEDICAL SOCIETY for the year 1881. J. H. Jerome, M.D., Saginaw, President; George E. Ranney, M.D., Lansing, Secretary.

This is a small volume of one hundred and two pages, containing only seven short papers in addition to the daily proceedings of the body, the president's address, reports of officers, committees, etc. Among the papers we note "The Cure of Acute Glaucoma by Sulphate of Eserine," by C. J. Lundy, M.D.; "The Treatment at Birth of Congenital Club-foot," by T. W. Reynolds, M.D.; "Cocculus Indica in Epilepsy," by C. B. Burr, M.D.; and "Retro-uterine, or Intra-peritoneal Hæmatocele," by I. E. Brown, M.D.

We think the arrangement of the volume would be improved by placing the list of officers, table of contents, etc., in the front of the book, and by observing a better order in the classification and location of the contents—the addresses, reports and voluntary papers.

---

Medical Communications of the Massachusetts Medical Society. Henry W. Williams, M. D., Boston, President; Francis W. Goss, M. D., Roxbury, Secretary.

The contents of this volume consist of two articles—"The annual discourse :—Medical Societies, their organization and the nature of their work," by J. Collins Warren, M. D., Boston, and "A Centennial Address," by Samuel Abbott Green, M D., Boston—and the regular proceedings of this, the centennial meeting.

The centennial dinner—including a poem by Dr. O. W. Holmes among the courses—receives a full share of attention. The treasurer's report is refreshing. From it we learn that the funded property of the society amounts to $31,420.31. The income for last year was $8,160.74. Expenses, $5,617.50. Balance carried over, $1,543.24. Among the expense items is $1,744.31 for the aforesaid dinner.

The salary of the treasurer is $400 per annum.

Financially, at least, the society may be said to be in a healthy condition.

---

Library of Medical Classics.

Emulating the example of the publishers of cheap editions of the lighter literature of the day, Messrs. Bermingham & Co., are publishing in pamphlet form, and at a very low cost, monographs on various subjects by well-known authors.

The parts are issued semi-monthly, at eight dollars a year. The price of individual numbers seems to be regulated by their size, which is not uniform. Four numbers have already appeared. They are : "A practical manual of the treatment of diseases of the rectum," by Henry Smith, F.R.C.S., London ; " Clinical lectures on the diseases of women, delivered in St. Bartholomew's hospital," by J. Mathews Duncan, M.D., LL.D., F.R.S.E., etc., London ; "A manual of veneral diseases, for students and practitioners," by Berkeley Hill and Arthur Cooper, and the admirable treatise by J. Milner Fothergill, M. D., on " Indigestion and biliousness."

